# Experimental Design for Parameter Estimation of Gene Regulatory Networks

**DOI:** 10.1371/journal.pone.0040052

**Published:** 2012-07-16

**Authors:** Bernhard Steiert, Andreas Raue, Jens Timmer, Clemens Kreutz

**Affiliations:** 1 Institute for Physics, University of Freiburg, Freiburg, Germany; 2 Freiburg Center for Systems Biology, University of Freiburg, Freiburg, Germany; 3 Freiburg Institute for Advanced Studies, University of Freiburg, Freiburg, Germany; 4 BIOSS Centre for Biological Signalling Studies, University of Freiburg, Freiburg, Germany; 5 Department of Clinical and Experimental Medicine, Linköping University, Linköping, Sweden; Mount Sinai School of Medicine, United States of America

## Abstract

Systems biology aims for building quantitative models to address unresolved issues in molecular biology. In order to describe the behavior of biological cells adequately, gene regulatory networks (GRNs) are intensively investigated. As the validity of models built for GRNs depends crucially on the kinetic rates, various methods have been developed to estimate these parameters from experimental data. For this purpose, it is favorable to choose the experimental conditions yielding maximal information. However, existing experimental design principles often rely on unfulfilled mathematical assumptions or become computationally demanding with growing model complexity. To solve this problem, we combined advanced methods for parameter and uncertainty estimation with experimental design considerations. As a showcase, we optimized three simulated GRNs in one of the challenges from the Dialogue for Reverse Engineering Assessment and Methods (DREAM). This article presents our approach, which was awarded the *best performing procedure* at the DREAM6 *Estimation of Model Parameters* challenge. For fast and reliable parameter estimation, local deterministic optimization of the likelihood was applied. We analyzed identifiability and precision of the estimates by calculating the profile likelihood. Furthermore, the profiles provided a way to uncover a selection of most informative experiments, from which the optimal one was chosen using additional criteria at every step of the design process. In conclusion, we provide a strategy for optimal experimental design and show its successful application on three highly nonlinear dynamic models. Although presented in the context of the GRNs to be inferred for the DREAM6 challenge, the approach is generic and applicable to most types of quantitative models in systems biology and other disciplines.

## Introduction

In a deterministic framework, the kinetics of gene regulatory networks (GRNs) can be described by ordinary differential equations (ODEs). The kinetic constants as well as the initial concentrations are usually unknown and have to be estimated from experiments. Furthermore, precise predictions of the systems’ behavior under perturbed conditions confirm the model reliability. To identify informative measurements out of all feasible experiments, parameter estimation and experimental design have to be applied iteratively to calibrate the model efficiently [1]. In this article, we present our methods and results that received the *best performer award* at the DREAM6 *Estimation of Model Parameters* challenge [2], [3]. The main aspects are the calculation of maximum likelihood estimates for parameters, determining confidence intervals by exploiting the profile likelihood [4], and performing experimental design to reduce the leading uncertainties of parameters and predictions.

## Methods

### Problem Definition

Finding the most informative experimental conditions is a highly nontrivial process. In general, it has to be decided at which time points the measurements are performed, what quantities are observed, and under which perturbations the data are obtained. The first step is to define an objective function specifying the purpose of the experimental design, e.g. the expected accuracy of estimated parameters and/or the expected accuracy of predictions of the model. Once this goal is fixed, different experimental designs can be scored.

**Figure 1:**
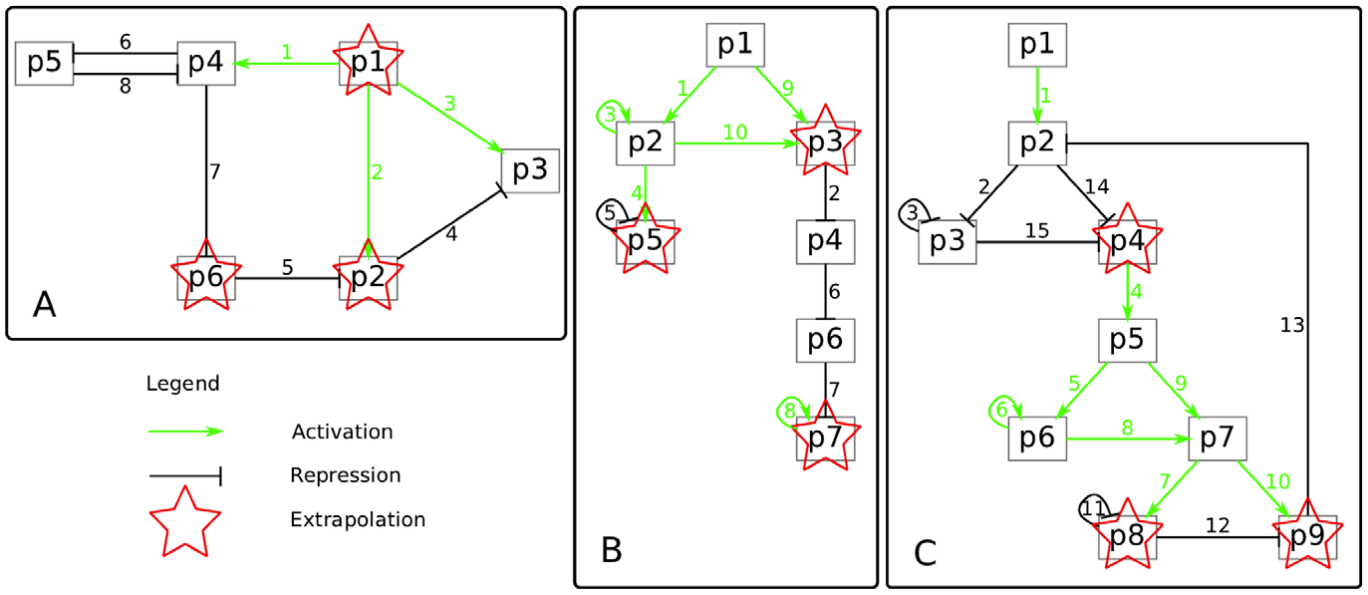
Models of the DREAM6 *Estimation of Model Parameters* challenge. The two steps of transcription and translation have been combined. Green arrows represent an activating interaction while black lines indicate a repression. Numbers on edges correspond to the numbering of the Hill kinetics used by the organizers of the challenge. The proteins marked by a red star have to be predicted under a perturbed setting to evaluate the accuracy of model predictions. A: Model 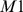 with 29 kinetic parameters and 8 Hill coefficients/interactions; B: Model 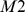 with 35 kinetic parameters and 10 Hill coefficients/interactions; C: Model 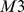 with 49 kinetic parameters and 15 Hill coefficients/interactions.

In the DREAM6 *Estimation of Model Parameters* challenge, the goal was to perform experimental design considerations for spending a virtual budget to estimate parameters of the gene regulatory networks and to be able to extrapolate the systems â€™ behavior, i.e. to predict the dynamics under a perturbed condition. The model parameters of three GRNs (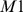, 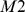 and 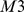) as shown in Fig. 1 had to be estimated. The model topologies, reaction rate equations and initial conditions were given, so the unknown features of the challenge were only the kinetic parameters. For activation and repression of genes, Hill kinetics were assumed. Protein production was modeled by a two step process, i.e. transcription and translation, as described in the following section.

In addition to wild-type experiments, three classes of perturbation experiments were considered, namely gene deletions, siRNA knock-down experiments, and changes of the ribosomal binding site activity. Time course measurements of mRNAs or proteins could be purchased. Every possible design had assigned virtual costs. A noticeable peculiarity was the possibility of directly measuring exact parameters, as discussed later in more detail. A detailed description of the models and further information about the whole challenge like the scoring scheme representing the objective function to rank the performance of the participants can be found in [3].

### Kinetic Equations

The dynamics
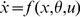
(1)of the molecular compounds or species *x* of the GRNs was given by first-order ODEs with parameters 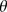 and perturbations *u*. Each component of *f* is a sum of reaction rates *v* of the respective species. For activation and repression, Hill kinetics were assumed that are represented by two parameters for each interaction, i.e. one Hill coefficient 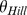 and a Kd-value 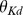, respectively. E.g. the transcription of the gene for protein *p*3 of model 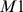 has the rate



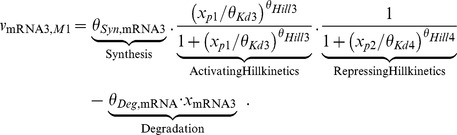
(2)If a protein is activated or repressed by more than one transcription factor, the corresponding rates were added up. Translation was modeled using mass-action kinetics, i.e. by a linear ODE
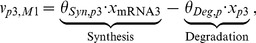
(3)where the synthesis parameters 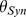 can be interpreted as the strengths of the ribosomal binding sites. Degradation rates 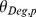 were assumed to be equal for all proteins of a model hence effectively only being one parameter. The degradation rates of the mRNAs 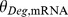 were set to 1 by the organizers and subsequently not investigated further. With these descriptions at hand, the networks visualized in Fig. 1 can be translated into kinetic models. The parameters of interest were from the following categories: production or degradation strengths, Hill coefficients, or Michaelis Menten constants 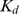. In total, there were 29 parameters to be estimated for model 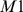, 35 for 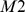, and 49 for 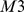, respectively.

In the challenge, gene deletion was regarded by elimination of the production of both, the targeted mRNA and protein, i.e. 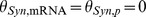. For siRNA perturbations, the corresponding degradation rates were changed by five fold, changes of a ribosomal binding site activity was incorporated by increasing 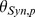 by a factor of two.

**Figure 2:**
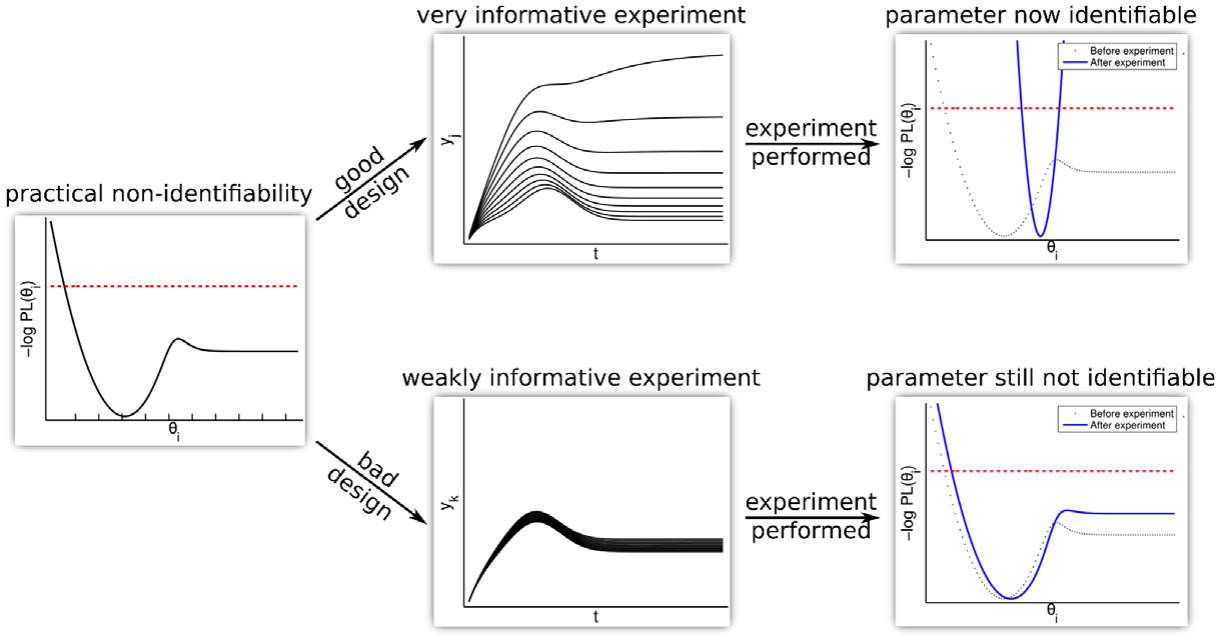
Experimental design procedure based on profile likelihood. The left panel shows the profile likelihood of a practically non-identifiable parameter. To resolve the corresponding uncertainty of the parameter estimate, a set of parameter vectors along its profile is chosen, represented by the dashes on the x-axis. The parameters are subsequently modified according to two designs, a highly informative experiment in the upper branch and a weakly informative experiment in the lower branch. Model predictions are simulated for the chosen parameter vectors, represented by the time courses in the middle column. A wide spread of the simulated time courses indicates an informative experiment. The impact of data purchasing is shown in the most right subplots by the blue curves, where the acquisition of informative data narrows the parameter down to a small variance, in contrast to a poor experiment with nearly no improvement of the estimate.

### Parameter Estimation

The parameters 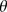, i.e. the kinetic rates, have been estimated by maximum likelihood. The likelihood
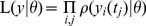
(4)is the joint probability of independently distributed measurements *y* of compound *i* at time point 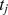 with each data-point distributed according to 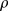. Each measurement is simulated using the error model
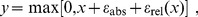
(5)where the absolute error 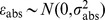 and the relative error 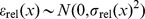 are Gaussian random variables with standard deviations 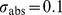 and 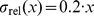. This error model represented by the parameters 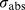 and 0.2 in the formula for 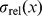 was provided by the organizers at the time the challenge was initiated.

The following considerations were done by the authors. In general, a sum 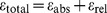 of two independent Gaussian random variables 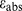 and 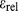 is again normally distributed 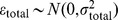 with variance
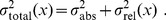
(6)


Therefore, the likelihood if no clipping of negative values to zero is present is
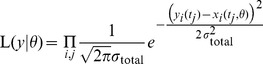
(7)with 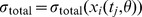.

According to (5), negative data realizations were clipped to zero which was accounted for in the likelihood by
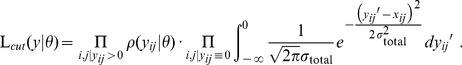
(8)where 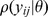 denotes the contribution of a single data point to the likelihood according to (7). The integral corresponds to the probability of negative data realizations for given 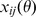.

**Table 1:** Overview of the criteria that were considered for the final decisions.

Abbreviaton	Detailed explanation
(WT)	Wild-type measurements provide the largest data-points to credits ratio.
(P> mRNA)	Protein data has a better data-points to credits ratio than mRNA data. However, the species to be measured have to be specified and choosing the wrong time-courses can yield only little information gain.
(MA)	For microarray data, there is no decision required about which compounds should be measured. This makes the design more robust. If there are fast processes, high-density time resolution is favorable in comparison to low-density measurements.
(OptPerPL)	Perturbation experiments *D* are selected as maximally informative based on the PL, if the score *R*(*D*) in (13) is optimal.
(GelShift)	Because a single time course data set is not informative enough to resolve the practical non-identifiability, this parameter was measured directly by a gel-shift experiment.
(Module)	The parameters to be bought are in a sub-module of bad estimates and therefore there is hope to improve identifiability of the whole module.
(LocMin)	If several local minima have been detected with similar agreement to the data, designs are chosen which optimally discriminate between the local minima.
(SwitchDyn)	The model shows qualitatively different dynamics and a perturbation is able to switch the model’s behavior.
(Extra)	The experiment or the parameter values are important for improving the accuracy of the demanded model extrapolation.
(Budget)	Sometimes, experiments are advantageous because the remaining credits allow a more flexible planning or the budget can be spent more comprehensively.

The optimization of the experimental design has been performed on the basis of the arguments provided in this table. The abbreviations are used to indicate the reasons for iteratively purchasing data for the three models.

Due to the effort that would have been needed to incorporate (8) into our existing fast C-algorithm of the optimization routine, we used (7) in the experimental design stage and implemented (8) in a more flexible MATLAB-code only for the final parameter estimation step, i.e. after the whole budget was spent. The likelihood (8) is correct for the challenge, as it is in accordance with the error model (5) that was used for the simulation of measurements. However, for real biological data, other error models are usually more appropriate which is briefly explained in the discussion. For numerical reasons, the likelihood was log-transformed and the log-likelihood is denoted as LL in the following. Since the DREAM6 scoring metric penalizes the deviation of the estimated parameters 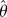 from the underlying truth, confidence intervals of the estimates were calculated to assess the accuracy of our estimates. For this purpose, the profile likelihood
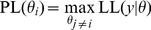
(9)for a parameter 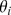 given the data *y* is utilized [4]. In (9), the optimization is performed for all parameters except 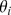. Confidence intervals for the estimation of a single parameter are then given by



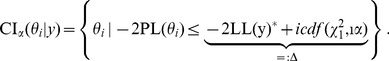
(10)Here, 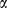 is the confidence level and 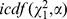 denotes the 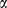 quantile of the chi-square distribution with one degree of freedom which is given by the respective inverse cumulative density function. 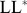 is the maximum of the log-likelihood function after all parameters are optimized. Every point on a profile 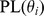 represents a corresponding parameter vector
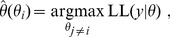
(11)containing the subsequently optimized remaining parameters. The profile likelihoods of all parameters imply a set
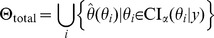
(12)of parameter vectors along all profiles and below the threshold 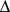, which are used for experimental planning. To identify experimental conditions, i.e. perturbations, for which the parameter uncertainties propagate into discriminable time-courses, a representative finite subset 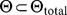 was chosen and the trajectories were simulated for every parameter vector 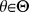. The perturbations were accounted for by adapting the respective parameter components of 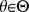 appropriately. A wide spread of the resulting trajectories indicated an informative experiment, promising a reduction of the uncertainty of the parameter values [5]. To measure the spread of the predictions, we calculated the rank
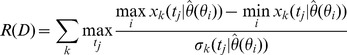
(13)of potential designs D, comprising measurements of species 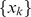 at finite time points 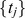 given by the experimental time resolution. Further possibilities for assessing the spread of predictions are given for example by the estimation of the variance on each time point or by substituting the maximization over time points by a summation in formula (13). However, other possible metrics to measure the spread yielded comparable results. The investigated ODE system yields non-linear solutions with respect to the parameters in general. Experimental design iteratively restricts the parameter space that is in agreement with the data. In an ‘asymptotic setting’, data quality and amount is sufficient to change the likelihood shape into a multivariate normal distribution. This is equivalent to the observation, that the trajectories 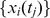 in LL have an almost linear dependency with respect to the parameters, yielding quadratic shaped LL and PL. The solution of the non-linear ODE system is therefore behaving locally linear in the parameter region where it is in statistical agreement with the data. Then, uncertainty can be translated in terms of standard errors or Fisher information. However, in non-asymptotic settings the non-linearity requires numerical heuristics for propagating uncertainties of parameter estimates to predictions.

The discussed experimental design procedure is visualized in Fig. 2. Here, the profile in the leftmost panel represents a practical non-identifiability, as its upper boundary is only given by the border of the parameter space. Two designs are evaluated by applying a perturbation, visualized in the upper and lower branches. To predict whether experiments are informative for identifying parameters, time courses are simulated for specific species under the given condition. An informative experiment results in a wide spread of predictions and the underlying sampling of 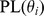 can be discriminated by acquiring the corresponding data, as can be seen in the upper branch of Fig. 2. In contrast, a weakly informative experiment is not able to resolve the non-identifiability, as shown in the lower branch. Here, the profile is still unrestricted to higher values and the uncertainty of the estimate remains large.

**Figure 3:**
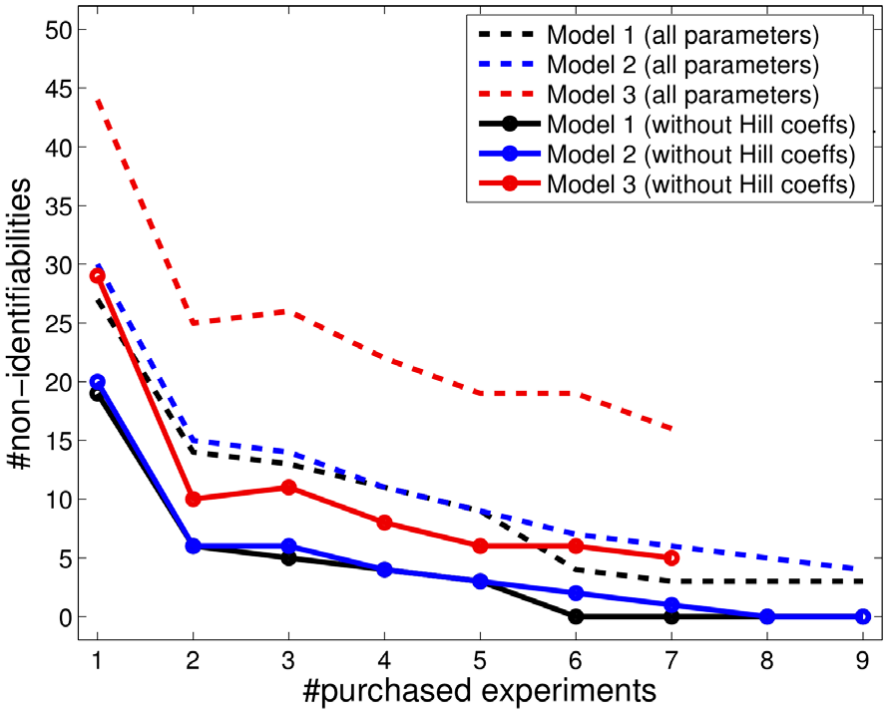
Number of practically non-identifiable parameters during the experimental design process. The elimination of non-identifiabilities was one of the major goals of the applied experimental design strategy. The number of practically non-identifiable parameters, represented by profile likelihoods reaching one or both borders of the parameter domain under a given statistical threshold, demonstrates the performance of the strategy (dashed lines). As the parameter domain for the Hill coefficients was restricted to a smaller range, i.e. 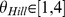, some of the underlying true values lay at the boundary of the parameter space. Counting the non-identifiabilities for Hill coefficients can therefore give a wrong impression when the MLE was correctly at the border of the parameter space. The number of practically non-identifiable parameters except the Hill coefficients is plotted by solid lines. Note that the number of non-identifiabilities is calculated from noisy data, hence the measurement errors propagate into these values. Therefore it can happen, that the number is increasing at some steps yet it is decreasing in general. An analogous observation can be made in Fig. 4.

The scoring metric to evaluate the performance of the participating groups at the DREAM6 *Estimation of Model Parameters* challenge consisted of two parts. The first was measuring the deviation of the estimated parameters from the underlying truth and the corresponding formula will be introduced in (14). The approach that was explained above and visualized in Fig. 2 optimized the expectation of this part of the score. The second part of the scoring metric penalized the deviation of extrapolated trajectories under certain given perturbations that were not accessible by experiments. In an analogous manner to the discussed selection of experiments, the influence of parameter uncertainties on the extrapolations has been studied by simulating the dynamics under the given prediction setting separately for each parameter along a finite subset of its profile, which yielded a set of extrapolations. Large variances of these predictions indicated parameter uncertainties with large impact on the desired extrapolations. Accordingly, the uncertainties of those parameters were analyzed with higher priority as discussed, i.e. finding the perturbations that maximized the spread of the experimental outcomes. In case of several experiments with similar performance, additional criteria were utilized which are discussed later.

**Figure 4:**
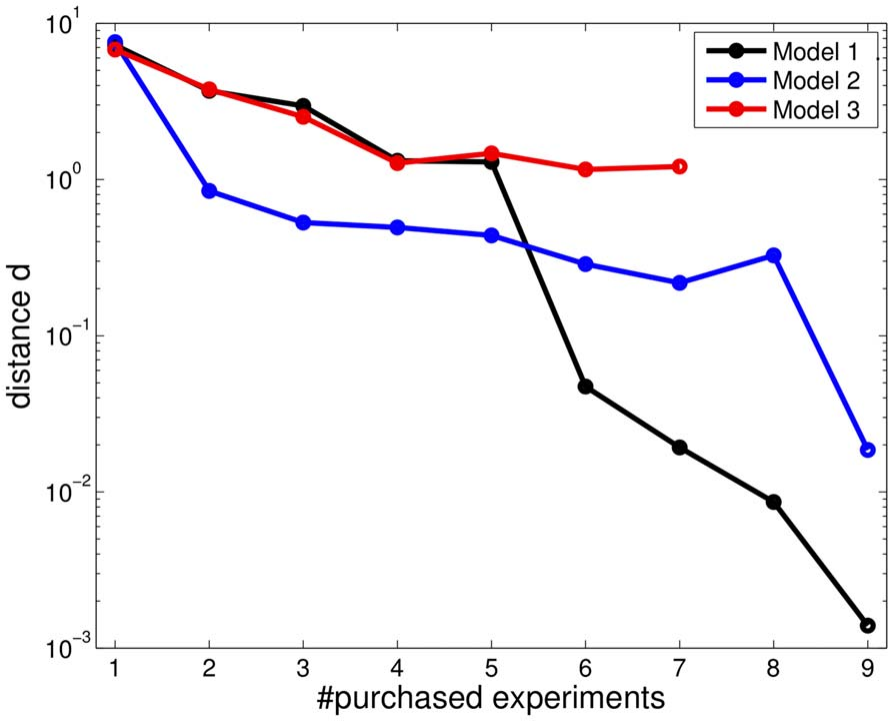
Deviation of true parameters and our estimates during the experimental design process. Purchasing informative experiments reduces the distance *d* of the estimates and the true parameters. Because the estimated parameters are random variables, the distance *d* sometimes increases by chance. This preferentially occurs if there are parameters with flat profiles.

**Table 2:** Summary of the decisions to spend the budget for model 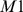.

Step	Action	Arguments	Remaining credits
1	Initial dataset		10000
2	WT protein data of *p*1–*p*6	**(WT)**,(P> mRNA)	8800
3	WT high-density MA	**(WT)**,(MA)	7800
4	*θ_Hill_*_1_,*θ_Kd_*_1_	**(GelShift)**	6200
5	*θ_Hill_*_3_,*θ_Kd_*_3_	**(GelShift)**	4600
6	siRNA mRNA5, measure *p*2&*p*4	**(OptPerPL)**,(Budget)	3850
7	*θ_Hill_*_2_,*θ_Kd_*_2_	**(GelShift)**	2250
8	rbs of *p*4, measure *p*2&*p*6	**(OptPerPL)**, (SwitchDyn), (Extra)	1400
9	siRNA mRNA5 high-density MA	**(OptPerPL)**, (Budget)	50

The arguments are provided in the order of their priority. If an argument dominated, it is displayed in bold-face.

### Optimization

For numerical optimization of the log-likelihood, the trust-region method [6] implemented in the MATLAB function LSQNONLIN was applied. The ODE system was solved by the CVODES algorithm [7]. For efficiency, local gradient and curvature information was considered. The calculation of derivatives of the LL with respect to the parameters is performed using the chain rule of differentiation. The sensitivities 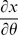 emerge as inner derivatives. For ODE systems, finite differences are not appropriate to approximate sensitivities [8]. Therefore, gradients are calculated from first order sensitivities obtained by integrating the sensitivity equations simultaneously to the ODEs for the dynamic variables [9]. We discovered numerical issues in solving the ODEs and sensitivity equations for model 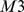 for initial conditions equals to zero and therefore we set the initial values of these compounds to 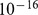.

To ensure that optimization yields global minima, multiple fits with latin hypercube sampling [10] of the initial guesses of the parameters have been performed. This means, that for 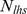 samples, the domain of each parameter component is subdivided into 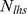 equally-sized intervals on the logarithmic scale. The vector samples are drawn so that each interval for each component is chosen exactly once. Within the intervals, the parameter components are drawn uniformly distributed. This procedure prevents the randomly drawn initial guesses to be accidentally close to each other.

**Figure 5:**
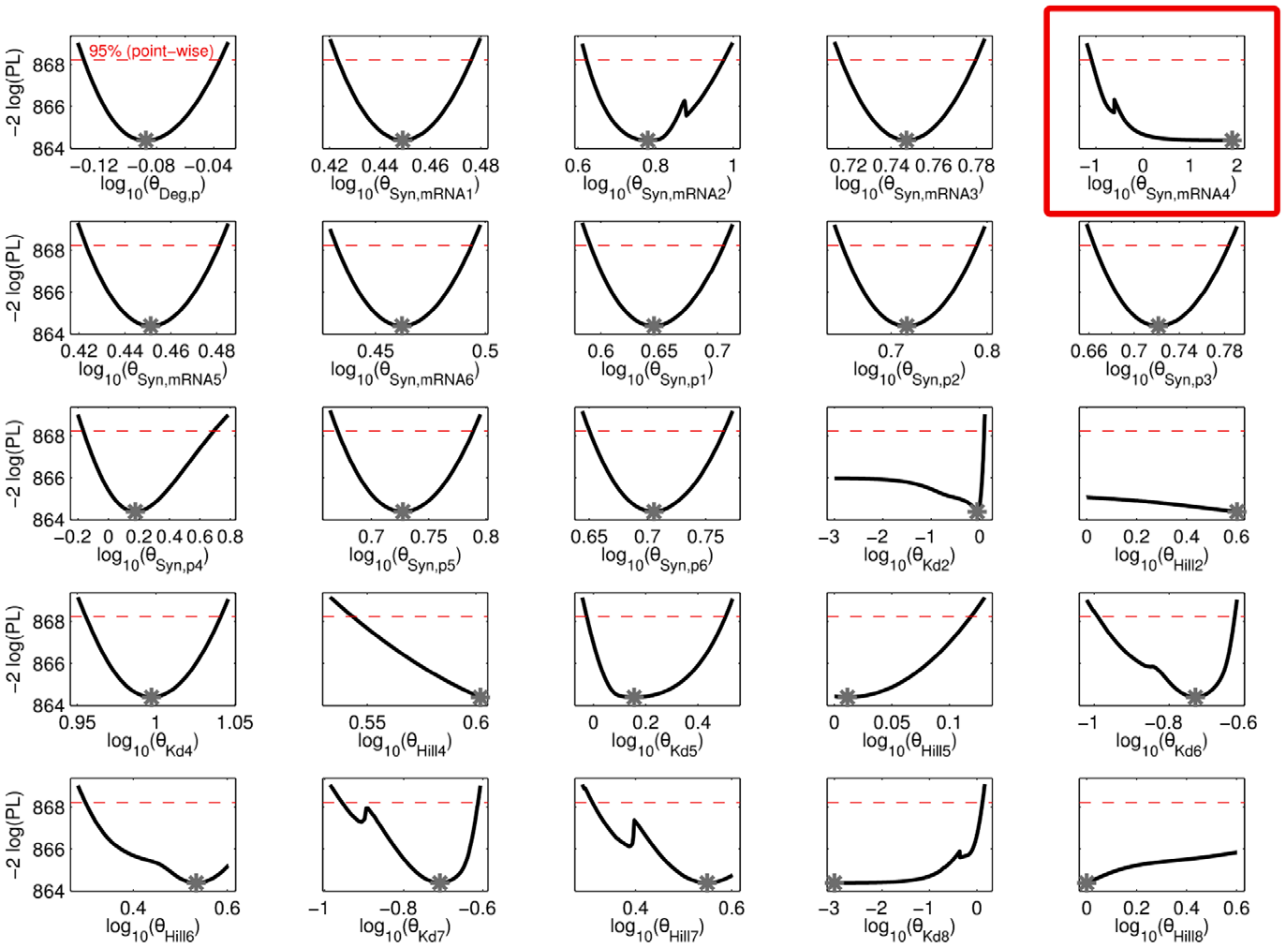
Profile likelihoods at step 5 in the experimental design process for model 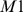. The marked profile for the production strength of mRNA4 shows a practical non-identifiability, since its upper boundary is only restricted by the border of the parameter space. Most parameters are already in an asymptotic setting, however some exhibit deviations from a quadratic profile likelihood.

**Figure 6:**
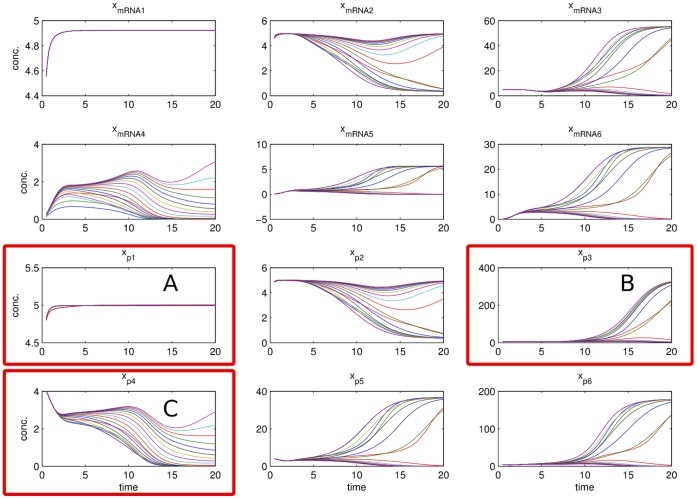
Trajectories of knock-down (siRNA) of mRNA5 experiment for model 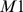 along the marked profile of Fig. 5. Three cases can be discriminated in this figure. In case A, there is almost no spread in the predictions and measuring protein *p*1 yields no additional information and represents a poor experiment. In case B, some spread can be seen in the predictions, but still many trajectories lying on top of each other. Hence, data acquisition of protein *p*3 for this perturbation is a medium informative experiment. In case C, a maximal informative experiment is visualized, represented by protein *p*4, that is able to accurately identify the parameter since the associated curves to every point on the profile are clearly discriminable. Note that the time courses have been normalized by corresponding standard deviation 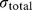, hence all trajectories of proteins start at 
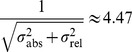
.

To decide whether several local minima sufficiently agree with the data, we used a threshold given by the log-likelihood of the best fit plus the 95%-quantile of the 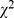-distribution with degrees of freedom equal to the number of parameters. This corresponds to a threshold utilized to calculate joint confidence intervals for all parameters. At the beginning of the experimental design process for all three models, we found roughly half of 1000 initial parameter combinations converging into the same optimum. The others either did not converge, since the initial values were in a region of the parameter space where the integrator failed to reach the demanded accuracy, or the optimizer converged in another local optimum. These needed to be discriminated during the design process. In the final stage of the experimental design setup, there was no second optimum below the statistical threshold, i.e. all further local optima yielded a highly improbable value of the objective function. This demonstrates that deterministic optimization combined with randomization of the initial values yields a good performance for global optimization in this application.

**Figure 7:**
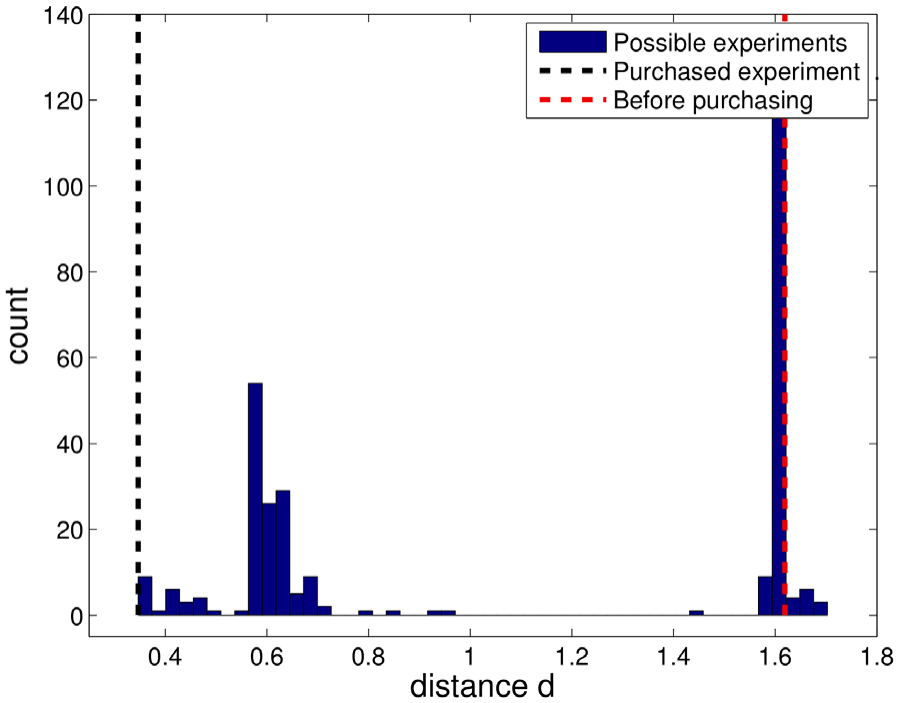
Comparison of all possible experiments at step 5 of the experimental design process of model 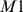. This figure demonstrates the information gain that could be obtained by purchasing time-course data. All possible experiments were taken into account. The smaller the distance (x-axis) after adding the data, the closer are the maximum likelihood estimates compared to the underlying truth. The purchased experiment was among the most informative. Moreover this particular experiment was cheaper than the other similarly informative experiments.

### Final Decisions

If the profile likelihood approach did not yield unambiguous suggestions, further criteria were taken into account for the final selection of each data acquisition. Table 1 provides a summary of such aspects.

Wild-type (WT) measurements for the mRNAs were provided in the start-up dataset, that was received by all participants. In general, WT data provided a quite large number of data points for less credits. Since it is advantageous to have measurements for all dynamic variables, we purchased WT measurements for all proteins as an initial step. This reduced non-identifiability issues and provided a minimal amount of information about the parameters allowing more detailed experimental design considerations.

The measurement of two proteins provided 80 data points for 400 credits, a high-density microarray experiments charged 1000 credits and yielded 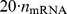 data points, i.e. 120, 140, and 180 for the three models. The ratio of the number of data points per credits was 0.2 for proteins and 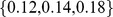 for RNA/microarray data for models 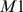, 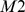, and 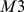. However, the larger the models, the more informative are microarray experiments in comparison to the information provided by the measurement of two proteins. Moreover, there is no decision required about which compounds should be measured. This makes the design more robust. If there are fast processes, high-density time resolution is favorable in comparison to low-density measurements.

To identify informative perturbation experiments, an objective function that ranks the information gain has to be introduced and to be maximized. We directly used the score (13) as objective function. At some steps, we performed a Monte-Carlo evaluation of a perturbation to confirm our guess. Practically non-identifiable parameters 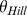 and 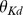 could also be measured directly by gel-shift experiments in contrast to the estimation from time-course data.

If considered parameters were in a sub-module of bad estimates, there was hope to improve identifiability of the whole module. Moreover, if several local minima were detected with similar agreement to the data, designs were chosen which optimally discriminate between the local minima. On the other hand, if the model showed qualitatively different dynamics, a perturbation is potentially able to switch the model’s behavior. For instance, oscillations can be diminished by influencing a negative feedback. For improving the accuracy of the demanded model extrapolations, the parameters influencing these predictions at most were calculated along the profiles and scored using (13). The uncertainty of the parameters that propagate into the extrapolations were then reduced in the discussed manner, as visualized in Fig. 2.

Sometimes, experiments were advantageous because the remaining credits allowed a more flexible planning or the budget could be spent in a way that enabled the authors to understand the model more comprehensively. E.g. siRNA experiments were the cheapest perturbations and provided often qualitatively the same information as a knockout. The saved credits could therefore be used to purchase further experiments under additional informative perturbations. For an objective argumentation, the cost of experiments could also be integrated (besides the score) to the objective function. As the cheapest perturbation experiment data-set charged 750 of the 10 k credits available for each model, we were also trying to optimize the eventually remaining credits to a low value. In practice, we tried to choose the experiments such that after the complete design process there were at most 200 credits left.

## Results

To account for strictly positive parameter values, all analyses have been performed in a logarithmic parameter space. Moreover, this accounts for the fact that changes of parameter values usually contribute multiplicatively rather than additively, i.e. changing a parameter by a factor *a* or 1/*a* has a similar impact, but adding a constant *a* has mostly a qualitatively different effect than the corresponding subtraction.

In our initial analyses, we restricted the parameter space to 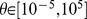. After some steps, we reduced this domain for numerical reasons, as most estimates were in agreement with 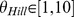 for the Hill coefficients and 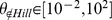 for the other parameters. However, this restriction was weakened again because the parameter estimation yielded values at the boundary of the parameter space and the profile likelihood indicated minima outside the parameter domain. For the final analyses, we used a continuous parameter domain of [1], [4] for Hill coefficients and 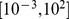 for other parameters. We were able to constrain the range for Hill coefficients more than for other parameters, which was first motivated from directly measured parameters by the gel-shift experiments. Additionally, the profiles supported this restriction, as they were either flat or in agreement with the interval [1], [4] for Hill coefficients.

The number of kinetic parameters were {29,35,49} for the three models. Keeping in mind the size of the parameter space, comprising roughly 5 orders of magnitude for each parameter, our final estimates were very close to the underlying truth that was provided by the organizers after the challenge: After the experimental design process, the mean deviation of the estimated parameters to the true values was 1.7% for 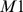, 5.2% for 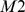, and 27.2% for 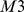.

### Successive Refinement of Scores

During the iterative experimental design process, we minimized the uncertainties of parameter estimates and model extrapolations. Non- or poorly identifiable parameters were tracked down to an asymptotic setting indicated by an almost quadratic profile likelihood. This is crucial, since not only the score of the final parameters and predictions could be optimized, but also the corresponding uncertainty. A non-identifiable parameter can be represented by a profile hitting one or both boundaries of the parameter space beneath the statistical threshold. As the major goal of the experimental design process is the estimation of model parameters and therefore the elimination of non-identifiabilities, the number of non-identifiable parameters demonstrates the performance of the applied strategy. This is shown in Fig. 3 by the dashed lines. The boundaries of the Hill coefficients were more restricted than for the other parameters, as discussed earlier. In this case, the underlying truth may equal a value at the boundary, hence creating a minimum of the corresponding profile at the border that represents an artificial non-identifiability. By neglecting the Hill coefficients, this number can be corrected, as visualized by the solid lines in Fig. 3. Our experimental design approach iteratively decreased the number of non-identifiabilities.

An alternative indication of the performance is given by the sum of the distances
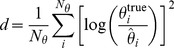
(14)of the maximum likelihood estimate (MLE) 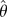 to the underlying parameters 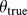, as can be seen in Fig. 4. The MLE can by chance be near the truth at an earlier stage of the design process, while still having large confidence intervals. The distance therefore does not have to be monotonously decreasing. In a real world application, this distance is not accessible in contrast to the shape of the profiles yielding a conclusion about the number of non-identifiabilities, as given in Fig. 3. Evaluating those distances is one of the advantages of simulated data, as given in the DREAM6 challenge.

An overview on the steps leading to our result on model 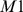 can be found in Table 2. To demonstrate the application of profile likelihoods on experimental design, one of the steps that were performed is discussed in detail here. Featuring many aspects of the experimental planning process, we chose step 5 to be examined.

As can be seen from the profiles in Fig. 5, the major non-identifiabilities represented by flat profiles have already been resolved. Nearly quadratic shaped profiles indicate the system to be in an almost asymptotic setting. However, e.g. the production strength of the mRNA4 showed a practical non-identifiability with a CI of approximately 3 orders of magnitude within the boundaries of the parameter space. The experimental design strategy based on profiles is demonstrated, using parameter sets 
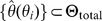
 along the profiles for predictions of each hypothetical experiments. The designs were ranked according to (13) and the best design is discussed in the following. Fig. 6 visualizes the time-course predictions of mRNAs and proteins for this perturbation, i.e. for the knock-down of mRNA5 by setting the parameter of the corresponding degradation rate 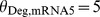. Three cases can be discriminated, representing weakly, medium and maximal informative compounds for this particular perturbation. The same procedure was applied for each combination of perturbations and species.

To show the influence of the choice of buying a particular experiment, we were able to compare all experiments after the underlying parameter values were provided. The influence of each data-set on the distance (14) is quantified in the following. Using model 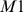 and the measurements available at step 5, a comparison of the performance of all possible decisions was implemented. As the assessment of parameter estimates depends on the noise realization, we simulated the experiments without error using the true parameters released after the challenge. To avoid the distortion of data weighting, the error model (6) was applied to the hypothetical measurements. After incorporating the additional experiments, the model was fitted to evaluate the improvement of the MLE. The distance given by (14), comparing the MLE with the underlying truth is calculated and visualized in the histogram in Fig. 7. In the leftmost bin, there are 9 highly informative experiments comprising our choice. Since the goal of this step was not only to improve the MLE, but also to resolve non-identifiabilites and to optimize the extrapolations while not using too many credits, our decision can be considered optimal.

The DREAM6 challenge asked not only for the parameter estimates, but as well for model extrapolations under experimentally not accessible perturbations. By plotting the extrapolations along the profiles, the sensitivity of the predictions on the parameters and their uncertainties was accessible. This was incorporated in the experimental design process by focusing on the parameters that had a dominating influence on the extrapolations. However, an unsatisfying spread in extrapolations always was related to non-identifiabilities. Therefore, no desperate emphasis on the predictions was necessary and resolving the identifiability issue subsequently reduced the extrapolation uncertainty. This supports our strategy to focus more on the estimation of model parameters, as presented in this article.

## Discussion

In this article, we summarized the experimental design strategy which was awarded the *best performer* at the DREAM6 *Estimation of Model Parameters* challenge. Optimally informative experiments had to be selected out of a set of feasible measurements. Our approach was based on the profile likelihood, which not only provides insights in parameter identifiability, but is as well useful for designing experiments. The uncertainty of the parameters was translated into uncertainty of the model predictions for the feasible experiments by forward evaluations of the ODEs. In this challenge, we had to chose between {331,516,1079} possible experiments for the three models. In general, forward evaluation of the model is much less time consuming than parameter estimation or other maximization procedures like optimization of design variables requiring ODE integration at each iteration. Therefore, the computational time of our approach is primarily given by the efforts of the profile likelihood calculation. The assessment of an experimental condition is quite efficient and therefore our approach is also feasible for more complex design issues which can occur for continuous design variables, e.g. for dose-response experiments. However, in reality there is also only a limited number of options possible in experiments, resulting in finite design variables. If the profile likelihood-based approach did not indicate the optimal experiment unambiguously, other arguments were incorporated for the optimal decisions. Although presented in the context of parameter estimation for GRNs, the approach is generic and applicable to most types of quantitative models in systems biology, e.g. signaling or metabolic networks.

The error model (5) that was used by the organizers to generate the data for this challenge assures non-negative values. Data being positive defined holds true for measurements of intensities, e.g. western blotting [11] or microarray [12] techniques. However, as shown in [13], a more realistic error model for these kinds of experiments is given by the log-normal distribution. After log-transformation of all trajectories and data, the errors are Gaussian distributed and negative values are allowed, as they are positive again after back-transforming via the exponential function. We therefore would not suggest to use the error model (5) for real applications.

In comparison to the simulated data and measurements of the challenge, further issues arise in a real world application. Usually not all molecular compounds in a network can be measured or only combinations of different species are accessible experimentally due to the specificity of antibodies. The ODEs then need to be extended by observation functions that represent those combinations. As total molecule numbers are often unknown, nuisance parameters that account for scaling of concentrations have to be introduced as well. For the challenge, gel-shift experiments corresponded to measurements without noise and the comparison of noisy time course data and exact parameter values was difficult. In practice however, direct parameter measurements exhibit uncertainty. Even though not accessible during the challenge, the presented strategies can be readily extended to different possibilities of measurement time points, which is a common question an experimentalist would ask.

For ODE models, calculating the uncertainty of model predictions always requires a sampling strategy of the parameter space. In this manuscript, the set of evaluated parameters was taken from the profile likelihood calculation. Alternative strategies comprise the prediction profile likelihood [14], as well as Markov chain Monte Carlo (MCMC) methods [15] and core predictions [16]. The efficiency of such sampling strategies in general strongly depends on the dimension and size of the parameter space. For a given dimension, i.e. number of parameters, the size of the space is specified by the admissible range of the individual parameters. In high-dimensional spaces, small changes of the admissible ranges have huge impact on the overall size. As an example, if the range is doubled for each parameter, the parameter space is enlarged by a factor of 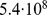 for model 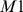, 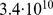 for model 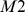, and 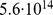 for model 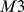. Therefore, a suitable choice of the parameter range can be decisive for the performance of the experimental planning. At the beginning of the challenge we allowed quite large ranges for the parameter values which had to be modified twice. In most cases, the likelihood profiles as well as the parameters we obtained by the gel-shift experiments indicated reasonable choices of the boundaries. Nevertheless, the choice can seriously impact the performance of experimental design strategies and therefore having some prior knowledge about the boundaries of the parameter space can be essential, especially if the performance of several approaches is intended to be compared.

Compared to the idealized situation in the DREAM6 *Estimation of Model Parameters* challenge, experimental possibilities in real world applications are much more limited. Consequently, experimental design becomes even more important for parameter estimation and for resolving non-identifiabilities in real applications. The experimental design approaches presented here were already applied successfully for the modeling of cellular signaling where they facilitated reliable parameter estimates and quantitative analysis of systems dynamics [17], [18]. However, the DREAM6 *Estimation of Model Parameters* challenge provided a very useful objective benchmark for judging and comparing the performance of parameter estimation and experimental design techniques. We could thereby demonstrate the performance of the methods we developed for systems biology applications.
